# The nature of Ordovician limestone-marl alternations in the Oslo-Asker District (Norway): witnesses of primary glacio-eustasy or diagenetic rhythms?

**DOI:** 10.1038/srep18787

**Published:** 2016-01-07

**Authors:** Chloé E. A. Amberg, Tim Collart, Wout Salenbien, Lisa M. Egger, Axel Munnecke, Arne T. Nielsen, Claude Monnet, Øyvind Hammer, Thijs R. A. Vandenbroucke

**Affiliations:** 1 UMR 8198 du CNRS: Evo-Eco-Paleo, Université de Lille – Sciences et Technologies, Avenue Paul Langevin, SN5, 59655 Villeneuve d’Ascq, France; 2 Department of Geology (WE13), Ghent University, Krijgslaan 281-S8, B-9000 Ghent, Belgium; 3 Nicholas School of Environment, Duke University, Environment Hall, 9 Circuit drive, Box 90328, Durham, NC 277708, USA; 4 FG Paläoumwelt, GeoZentrum Nordbayern, Friedrich-Alexander University of Erlangen-Nuremberg, Loewenichstr. 28, D-91054 Erlangen, Germany; 5 Paleoenvironmental Dynamics Group Institute of Earth Sciences, University of Heidelberg, Im Neuenheimer Feld 234, D-69120 Heidelberg, Germany; 6 Natural History Museum of Denmark (Geological Museum), University of Copenhagen, Øster Voldgade 5–7, DK-1350 Copenhagen K, Denmark; 7 Natural History Museum, University of Oslo, Sars gate 1, 0318 Oslo, Norway

## Abstract

Ordovician limestone-marl alternations in the Oslo-Asker District have been interpreted as signaling glacio-eustatic lowstands, which would support a prolonged “Early Palaeozoic Icehouse”. However, these rhythmites could alternatively reflect differential diagenesis, without sedimentary trigger. Here, we test both hypotheses through one Darriwilian and three Katian sections. Our methodology consists of a bed-by-bed analysis of palynological (chitinozoan) and geochemical (XRF) data, to evaluate whether the limestone/marl couplets reflect an original cyclic signal. The results reveal similar palynomorph assemblages in limestones and marls. Exceptions, which could be interpreted as reflecting palaeoclimatological fluctuations, exist at the species level: *Ancyrochitina bornholmensis* seems to be more abundant in the marl samples from the lower Frognerkilen Formation on Nakkholmen Island. However, these rare cases where chitinozoans differ between limestone/marl facies are deemed insufficient for the identification of original cyclicity. The geochemical data show a near-perfect correlation between insoluble elements in the limestone and the marls, which indicates a similar composition of the potential precursor sediment, also in the Frognerkilen Formation. This is consistent with the palynological data. Although an original cyclic pattern could still be recorded by other, uninvestigated parameters, our palaeontological and geochemical data combined do not support the presence of such a signal.

The Ordovician (485–443 Ma) has long been known as a greenhouse period, with a short glaciation during the Hirnantian age (c. 444 Ma)^1^. This terminal Ordovician glaciation coincided with the first of the “Big Five” mass extinction events of the Phanerozoic^2^. An emerging body of evidence now suggests that this global cooling and the onset of the “Early Palaeozoic Icehouse” (EPI) may have started much earlier than previously assumed^3^, i.e. during the early Katian^4^,^5^,^6^, before the Sandbian^7^ or even during the Early-Middle Ordovician^3^. This has fundamental importance, because an early phase of cooling could provide a driving mechanism for the major changes in biodiversity during the Great Ordovician Biodiversification Event (GOBE)^3^.

As compelling as these new ideas are, they remain to be tested. So far, the studies in support of a protracted EPI comprise: (1) δ^18^O data derived from conodont apatite at equatorial palaeolatitudes^3^ suggesting that the tropical sea surface temperatures (SST) cooled during Early-Middle Ordovician, to stabilize close to modern values of SSTs; (2) the sequence stratigraphic architecture of various Ordovician packages, such as those treated herein^8^, the Peninsula Formation (Floian to Darriwilian) in South Africa^9^ and the Darriwilian Hiswah and Dubaydib formations in Jordan^10^, which have been suggested to record 3^rd^ and 4^th^ order sea level changes corresponding to glacio-eustatic cycles; (3) microfossil evidence with relatively steep graptolite and chitinozoan equator-to-pole faunal gradients that suggest cooling towards the Hirnantian glacial maximum was already underway in the Sandbian^7^,^11^; and (4) General Circulation Models (GCMs) for the Early-Middle Ordovician^12^ that suggest a long-term cooling trend through the Ordovician. However, each of these methodologies is inconclusive on its own. Climate proxy data that are limited to the low palaeolatitudes may not be the best recorder of global climate^13^. Ordovician sea level variations are difficult to quantify and often are subject to complex facies interpretations and dating issues. Biogeographical studies of graptolites and chitinozoans, although potentially conclusive for the Late Ordovician, do not yet exist for the Early and Middle Ordovician. Furthermore, the degree to which GCM output approximates real palaeoclimates is limited by the accuracy with which we can quantify the prevailing boundary conditions in deep-time. The aim of this study is to discuss the nature of the Middle-Late Ordovician background climate by scrutinizing some of the stratigraphical evidence.

The large-scale alternation between limestone and marl dominated formations is one of the main features observed in the Ordovician succession of the Oslo-Asker District in Norway^14^. According to Nielsen^8^, these alternating formations are the result of changes in depositional depth, potentially representing lower order glacio-eustatic sea level fluctuations. The calcareous formations often are composed of, or include, decimeter scale alternations of limestones and marls. Such fine-grained calcareous rhythmites appear in large epeiric seas through the entire Phanerozoic^15^,^16^,^17^. The rhythmites in the Oslo-Asker District consist of uncompacted continuous or nodular limestone beds separated by marl (or shale, which is a field-descriptive term and has no meaning in terms of absolute carbonate content) interlayers that are strongly compacted. Transitions between continuous limestone beds and isolated nodular limestone in a marly matrix are observed throughout the succession. The origin of the limestone nodules in the Oslo-Asker District has been long debated. Some authors considered that the nodules formed by dissolution of continuous carbonate layers exposed to undersaturated sea-water^18^. Others argued that the nodular limestone are early diagenetic concretions formed by carbonate precipitation in pore space underneath the water-sediment interface^19^, eventually collating into limestone beds. The latter interpretation is supported by the majority of authors^20^,^21^,^22^. Yet other scenarios^23^ consider that the nodules are the result of bacterial activity where the sulfate reduction and fermentation by the bacteria led to the production of bicarbonate and sulfide eventually turned into carbonate and pyrite, commonly found in the rhythmites.

In Nielsen’s model^8^, these rhythmites, internally, may represent a higher order palaeoclimatological control, in turn driven by orbital forcing. Others argue that such lithologies could be the result of differential diagenesis, i.e., a process of redistribution of calcium carbonate from marl layers to emergent limestone beds by dissolution, migration, and re-precipitation of ions^24^ regardless of the presence or absence of primary sedimentary rhythms^17^,^25^,^26^,^27^. In this scenario, aragonite dissolves in the shallow marine burial environment and eventually cements the uncompacted limestone beds^24^,^28^.

Many of the Ordovician Oslo-Asker District rhythmites, if not all, are characterized by large positive excursions in δ^13^C values, as evidenced by data from the Frognerkilen^6^, Solvang^6^ and Skogerholmen (unpubl. data) formations in the Oslo Region and other districts. Bergström *et al.*^6^ identified the excursions in the Frognerkilen and Solvang formations as the Guttenberg Isotope Carbone Excursion (GICE) and Kope (or Rakvere) excursions, respectively. The systematic coincidence between rhythmites and stable isotope excursions suggest that the rhythmites have been deposited under different environmental conditions than the intertonguing dark shale formations, and may thus well represent packages deposited during times of lower order glacio-eustatic lowstands as suggested by Nielsen^8^. Alternatively, one could argue that the environmental conditions that caused the isotope excursions also were responsible for differential diagenesis and the production of rhythmites in those particular intervals: for instance, aragonite is necessary as source to fuel differential diagenesis^29^, and aragonite is mostly produced on and exported from shallow-water platforms during sea-level high-stands (“Highstand Shedding”)^30^,^31^. The times of peak isotope values may thus well have been times of aragonite production in the Oslo Region, hypothetically driven by changes in water composition (more oligotrophic) or sea-level (in this case, a relative increase). In this context, and even for the well-documented Hirnantian isotope excursion, it is debated whether the δ^13^C peak values correspond to peak glaciation^32^. In summary, although the isotope excursions suggest changes in the palaeo-environment, they do not exclude a diagenetic origin for the limestone/marl couplets.

To investigate the depositional conditions of the Oslo rhythmites, we conducted bed-by-bed analyses using palynomorph assemblages (chitinozoans), which are diagenetically inert compounds, combined with x-ray fluorescence (XRF) measurements of insoluble oxides and elements. An original cyclic signal should be reflected in the chitinozoan microfauna, as they are thought to have a planktic mode of distribution and characterize latitudinally restricted water masses that are inferred to be SST-controlled and as such track episodes of major climate change, much like modern zooplankton^11^,^33^. We should thus observe different palynomorph assemblages for the two lithologies, if the rhythmites are reflecting a primary environmental signal. Palynomorph assemblages are relatively robust against the effects of differential diagenesis, unlike calcareous microfossils that might be destroyed in the marl interlayers by dissolution and compaction^26^. However, they do suffer compaction in marls, obscuring some of their morphological characters, in comparison to their limestone-hosted counterparts^34^. Likewise, when plotting the diagenetically stable oxides and elements percentages and their linear fit, we should observe two distinct populations for the two lithologies, with each regression line having a different slope, pointing to different ratios of these constituents in the precursor sediments of limestones and marls and thus to an original cyclic signal^26^. The most suitable relation to detect this signal is TiO_2_/Al_2_O_3_. However, if such a systematic difference is not observed, i.e., the two populations plot along a similar or equal trend line, the rhythmites can be either a diagenetic enhancement of a primary rhythm, with original differences in parameters that have not been measured or have been destroyed during diagenesis (e.g. primary porosity, permeability, TOC content), or are entirely diagenetic^26^. This inability to demonstrate unambiguously a diagenetic origin is referred to as the “diagenetic dilemma”^26^.

We sampled four separate intervals of alternating limestone and marls in different outcrops in the Oslo-Asker District (Fig. 1), i.e., the Lysaker Member of the Huk Formation (Darriwilian), the transition between the Arnestad and the Frognerkilen formations (Sandbian-Katian), the Solvang Formation (lower Katian), and the Hovedøya Member of the Spannslokket Formation (upper Katian) (Fig. 2). A summary of the stratigraphy and sampled sections is presented in the methods section. If the oldest marl-limestone rhythmite in the Oslo-Asker District, the Huk Formation, proves to be an expression of glacioeustasy, this would correlate well with the postulated stabilization of a cool climate by the Middle Ordovician as suggested by Trotter *et al.*^3^, or with the early cold snap of Turner *et al.*^9^, and would become an important argument for a protracted Ordovician icehouse condition.

## Results

In total, 77 samples have been investigated for palynology and 7062 specimens of chitinozoans are recorded (Fig. 3). The section on Hovedøya Island yields the best preserved specimens (Fig. 4) whereas the sections at Vollen, Bygdøy and on Nakkholmen Island contain rather poorly preserved specimens, which hampers identification. This is likely due to the presence of Permian intrusions in the area^14^, and, notably for the Huk Formation, the tectonic deformation of the sediments. Differential preservation is also a factor, as the specimens found in limestones are better preserved than the ones found in marls. Sampled sections on Bygdøy and Nakkholmen Island yielded a higher amount of chitinozoans, in general about four specimens per gram of rock, whereas the sections from Vollen and Hovedøya Island yielded only half as many. The results of the palynological (see also Supplementary Figs S1, S3, S5, S7) and XRF analyses (Supplementary Figs S2, S4, S6, S8) are summarized for each stratigraphic interval.

### Huk Formation

Sixty samples were collected in the Lysaker Member of the Huk Formation. A first batch was almost barren, and only 22 samples from the second batch, yielding a significant amount of chitinozoans, were retained for further analyses. They yielded 1394 chitinozoans in total, of which 891 were identified to the species level. Five genera and eight species were distinguished in this lowly diverse section (Fig. 3a). *Cyathochitina* is the most abundant genus (73% of the assemblage). It is present throughout the section but becomes increasingly abundant towards the top. The other genera with a marked abundance are *Conochitina, Desmochitina, Lagenochitina* and *Rhabdochitina*. Their abundance is low but constant through the section. The HCA (Supplementary Fig. S1) shows a complete intermixing of the chitinozoan taxonomic composition between the two lithologies. The DCA (Supplementary Fig. S1) also shows a strong overlap of the taxonomic components between the different lithologies, suggesting a rather similar chitinozoan composition between limestone and marl. Furthermore, the ANOSIM (R = −0.06; *p* = 0.812) and PERMANOVA (Pseudo-*F* = 0.06; *p* = 0.535) both show that chitinozoan assemblages are not significantly different between the two lithologies for the entire section. The most abundant species are *Cyathochitina calix* and *Cy. campanulaeformis* (Fig. 4.2). In the lower part of the section *Cy. calix* dominates, and *Cy. campanulaeformis* progressively appears and finally becomes more abundant towards the upper part of the section. The species for the other genera are left in open nomenclature (as ‘sp.’) due to poor preservation. Similar to the analyses at the genus level, we observe no lithology-specific assemblages at the species level, as shown by the HCA and DCA (Supplementary Fig. S1); the ANOSIM (R = 0.03; *p* = 0.294) and PERMANOVA (Pseudo-*F* = 0.10; *p* = 0.233) confirm this result.

The same 22 samples were used for XRF analysis. The bivariate scatter plots of TiO_2_ and Al_2_O_3_ percentages show two groups representing the limestones and marls. Their regression lines show no significant difference in slope (*p* = 0.179, Fig. 5a). This absence of significant difference in slopes between the lithologies is confirmed by the data for the other elements and oxides that are preferentially bound to clay minerals (K_2_O, SiO_2_, and Rb), the data for the oxides and elements bound to both clay and calcite minerals (Fe_2_O_3_, Zn, MgO), and the data for the elements and oxides bound only to calcite minerals (Sr), except for the MnO values, which show a significant (*p* = 0.035) difference in slopes between the lithologies. All the charts are uploaded as supplementary material (Supplementary Fig. S2).

### Arnestad/Frognerkilen formations

Of the 34 samples collected, 24 contain a significant number of palynomorphs. In total, 2712 specimens were identified to 12 genera and 22 species (Fig. 3b). The genus *Spinachitina* is the most abundant throughout the section, comprising 57% of the assemblage, followed by *Conochitina, Ancyrochitina, Belonechitina*, and *Desmochitina* with much lower and sporadic abundances (respectively, 18%, 9%, 8% and 6%). The other genera present have a very low relative abundance and are not considered (see Fig. 3b). The HCA and DCA show that there is no clear separation between the two lithologies regarding assemblage composition at the genus level throughout the section (Fig. 6). Furthermore, ANOSIM (R = 0.12; *p* = 0.029) and PERMANOVA (Pseudo-*F* = 0.15; *p* = 0.012) confirm that the chitinozoan assemblage is not influenced by the lithology (Fig. 6). Although these two tests indicate a significant difference (*p* = 0.029) in composition between limestone and marl, the R value of the ANOSIM indicates that the groups are barely distinguishable (R = 0.12). This pattern indicates that one or a few taxa within the assemblage may have different abundances between lithologies. The ANOVA performed on each taxon separately (Supplementary Fig. S3) indicates that only one genus has a significant difference in mean abundance between the two lithologies (F = 6.21; *P* = 0.015): *Ancyrochitina* thus has a lower relative abundance in limestones and a higher relative abundance in marls. Interestingly, the genus *Conochitina* has a different relative abundance, although this is not significant (F = 3.25; *p* = 0.075). In contrast, the genus *Spinachitina*, which is the most abundant, is clearly not affected by the lithology (F = 0.04; *p* = 0.840).

At species level, *Spinachitina multiradiata* (Fig. 4) is the most abundant (35%) and ranges throughout the section. The second most abundant species is *Conochitina* sp. with 18%. We observe a biostratigraphic separation between the upper part of the section, corresponding to the Frognerkilen Formation, and the lower part of the section, corresponding to the Arnestad Formation: *S. cervicornis* (Fig. 4) is abundant in the latter formation and decreases towards the top (Fig. 3) while *Ancyrochitina bornholmensis* and *Belonechitina robusta* (Fig. 4) become more abundant. Much as observed at the genus level, the HCA and DCA show no clear separation between the two lithologies regarding assemblage composition at the species level throughout the entire section (Fig. 6). Similarly, ANOSIM (R = 0.11; *p* = 0.049) and PERMANOVA (Pseudo-*F* = 0.11; *p* = 0.023) confirm that chitinozoan assemblages are not generally influenced by the lithology, bar perhaps a few taxa. The ANOVA (Supplementary Fig. S4) indicates that only one species has a significant difference in mean abundance between the two lithologies (F = 4.19; *p* = 0.044), i.e., *A. bornholmensis.* Again, the most abundant taxon, *Spinachitina multiradiata*, does not display a significantly different relative abundance between lithologies (F = 0.27; *p* = 0.869).

Out of the 24 samples analyzed for palynology, 15 were used for XRF. As for the Huk Fm., the slopes of the regression lines from limestones and marls are not significantly different for TiO_2_/Al_2_O_3_ (*p* = 0.126, Fig. 5b), as also observed for K_2_O, but the other elements and oxides bound to clay minerals present a significant difference (*p*(SiO_2_) = 0.009, *p*(Rb) = 0.003) between the limestones and marls. The elements bound to both clay and calcite minerals display no difference between the limestone group and the marl group for Zn and Fe_2_O_3_, after removing two outliers of Fe_2_O_3_ in samples TC 12-226 and TC 12-203, but the trend lines from MgO present a significant difference (*p* = 0.035) between the lithologies. The elements bound to calcite minerals show a significant difference in slopes for Sr (p = 0.047), when two peak values of Sr from the samples TC 12-226 and TC 12-203 were removed, but not for MnO (Supplementary Fig. S4).

### Solvang Formation

In this section, 18 of the samples collected yielded palynomorphs. Out of 1612 chitinozoans found, 1535 specimens were successfully identified to the genus level and 1209 specimens down to species level (Fig. 3c). In total, 11 different genera have been encountered and 16 different species were identified with confidence. *Belonechitina* is the most abundant genus (56% of the assemblage) and *Cyathochitina* is the second most abundant, representing 20% of the assemblage. The genus *Spinachitina* is moderately abundant (7%) and is only present in the four first samples of the lower part of the section (WSA 12-101B, WSA 12-102, WSA 12-030B, WSA 12-031), where the overall abundance and diversity of palynomorphs are low. Other genera occur in low abundance (Fig. 3c). All the genera are equally present in limestones and marls as shown by the HCA, DCA, ANOSIM (R = −0.05; *p* = 0.684) and PERMANOVA (Pseudo-*F* = 0.03; *p* = 0.647) (Supplementary Fig. S5). At the species level, the *Belonechitina hirsuta* complex (Fig. 4) is the most abundant ‘species’^35^ (27%). The second most abundant species is *Belonechitina robusta* (Fig. 4) representing 23% of the assemblage. *Cyathochitina campanulaeformis* and *C. kuckersiana* (Fig. 4) have been grouped together as their onerous differentiation mainly revolves around the length of a fragile carina. They are present throughout the section with a low relative abundance. The species *Spinachitina bulmani* and *Spinachitina multiradiata* are found only in the four basal samples, which clearly separates them from the rest of the section. At the species level, there is no evidence of lithofacies control on the palynomorph assemblages as suggested by the HCA, DCA, ANOSIM (R = −0.01; *p* = 0.443) and PERMANOVA (Pseudo-*F* = 0.05; *p* = 0.516) (Supplementary Fig. S6).

The same micropalaeontology samples were used for XRF except for the lowest two: WSA 12-101B and WSA 12-102. Once again, the slopes of the trend lines of both limestone and marls are similar for TiO_2_/Al_2_O_3_ (*p* = 0.862, Fig. 5c), thus showing no difference in slopes between the two lithologies, nor do the other elements bound to clay minerals (K_2_O, SiO_2_). The Rb data, when removing an outlier from the sample WSA-074A, are an exception, displaying a significant difference in slopes (*p* = 0.029) There is no difference in slopes for both lithologies in the elements included in both crystal lattices of calcite and clay minerals such as Zn and Fe_2_O_3,_ even when removing two peak values of Fe_2_O_3_ from WSA-12-090 and WSA-12-074A, but MgO present a significant difference in slopes of *p* = 0.0003 between the limestones and the marls. The elements only bound to calcite minerals present a significant difference in slopes for Sr (*p* = 0.001), but not for MnO. (Supplementary Fig. S6).

### Skogerholmen Formation

Out of 31 samples collected from this section, 21 yielded sufficient amounts of palynomorphs. In total, 2445 specimens of chitinozoans were identified, and 10 genera were encountered representing 13 species (Fig. 3d). In the Hovedøya Section, *Cyathochitina* is the most common genus (25%) and it is found throughout the section although in very variable abundancies. The second most abundant genus is *Belonechitina* (24%). It is also present throughout the section but its concentration is rather constant, except in the upper part where it becomes rare. The genus *Desmochitina* comprises 16% of the assemblage. Its abundance in the section is very variable, half of the total population being found in three samples. The genus *Spinachitina* (13%) is found almost exclusively in the upper part of the section, except for five specimens scattered in the lower part of the section. Similar to the three other intervals studied, the HCA and DCA reveal no specific grouping of palynomorphs between marls and limestones at the genus level, and the statistical analysis is in agreement (ANOSIM: R = −0.06; *p* = 0.737; PERMANOVA: Pseudo-*F* = 0.01; *p* = 0.925), indicating that the lithology has no influence on the assemblages (Supplementary Fig. S8). At the species level, the most abundant species are *Cyathochitina kuckersiana* and *C. campanulaeformis*, grouped together (25%). *Belonechitina micracantha* and *B. gamachiana* (Fig. 4) are rather abundant (13% and 12% of the assemblage, respectively). *Belonechitina micracantha* appears when *B. gamachiana* disappears. The species *Spinachitina multiradiata* appears in the lower part of the section with only five specimens and becomes relatively abundant (12%) in the upper part of the section. *Desmochitina ovulum* is moderately abundant (11%) throughout the section. *Spinachitina* cf. *taugourdeaui* (Fig. 4) has a very low abundance, but is remarkable as a biostratigraphical index species of the basal Hirnantian or Porkuni Baltic stage. The confirmation of this species, here in open nomenclature, would be important for the further interpretation of this section. Much like at the genus level, the HCA, DCA, ANOSIM (R = −0.07; *p* = 0.833) and PERMANOVA (Pseudo-*F* = 0.01; *p* = 0.932) (Supplementary Fig. S8) show no significant difference in species assemblages between the two lithologies.

The same 21 samples were used for XRF. As in the sections discussed above, the two lithologies present no significant difference in their regression slopes for TiO_2_/Al_2_O_3_ (*p* = 0.397, Fig. 5d), as for the other elements and oxides bound to clay minerals (Rb, SiO_2_), except for the K_2_O data that show a significant difference in slope (*p* = 0.016) between the limestones and marls. The elements and oxides that can be included both in the calcite lattice and in clay minerals present a relatively similar trend line with no significant difference in slopes between both lithologies for MgO and Fe_2_O_3_. Zn is the exception, where the trend lines of limestones and marls are significantly different (*p* = 0.015), and the same applies for the elements and oxides bound to calcite minerals (*p*(Sr) = 0.015, *p*(MnO)= 0.0003). (Supplementary Fig. S9).

## Discussion

In the four intervals studied, the assemblages of chitinozoans are generally independent of the lithology sampled (in terms of relative abundance). The HCA and DCA fail to demonstrate a clear separation in composition of assemblages between the limestones and the marls. The ANOSIM and PERMANOVA confirm this result, with very low R values between −0.10 to 0.15 indicating no difference between the lithologies, except for the Nakkholmen section. All these methods indicate that the lithology does not control the total taxonomic composition of the samples. However, one exception is observed: the ANOVA reveals that the genus *Ancyrochitina* and the species *Ancyrochitina bornholmensis*, found in the Arnestad/Frognerkilen Formation interval, are significantly more abundant in the marls. It is important to note that the ability to accurately identify species sometimes depends on differential preservation in certain lithologies: limestones, for instance, notably allow for fine details and relief to be preserved, where as specimens in marls are often flattened and fine ornamental details destroyed. The identification of *A. bornholmensis*, however, does not rely on such vulnerable criteria, suggesting that the systematic difference in relative abundance of this species could reflect primary differences. Such a signal could support Nielsen^8^ who interpreted the Frognerkilen Formation as a lowstand event, identified by Bergström^6^,^36^ as the GICE, one of the first glacial episodes in the EPI^4^,^37^. However, the cyclic variation of only one species amongst many, in a very restricted part of the section (10 samples) remains unconvincing.

Another potential problem linked to differential preservation is observed in the lower part of Skogerholmen Formation, relating to the species *Belonechitina gamachiana* and how it appears in limestones and marls. The shape and size of the specimens are the same, but the specimens from limestones have long basal spines combined with short ones along the chamber, whereas in marls they just carry a few remains of spines and often appear almost glabrous.

To identify a primary signal from the geochemical XRF analyses from the rhythmites, we should expect two trend lines with different slopes for the limestones and marls when plotting the diagenetically stable trace elements and oxides^38^. In all of our four intervals, the TiO_2_/Al_2_O_3_ data, which is the most suitable to detect a primary difference in the sediment, show no significant difference in the slopes of the trend lines between the limestones and marls. The other oxides and elements bound to clay minerals present more contrasted results in all the intervals except for the Huk Formation, where we found no difference in slopes for the four elements and oxides tested: The Arnestad/Frognerkilen formations and the Hovedøya Member display significant differences in slopes for K_2_O. The Arnestad/Frognerkilen formations and the Solvang Formation also show significant differences in slopes for Rb. These differences in slopes can be explained either by the fact that the Huk Formation is the most diagenetically overprinted section, or because the three other sections had slight differences in their precursor sediments. The elements and oxides that can be included in both the calcite lattice and in clay minerals display mixed results as well as the elements and oxides only bound to carbonates. This is mainly due to the high dispersion of the data. The overall results are in good agreement with the process of carbonate redistribution by differential diagenesis. The high correlation of diagenetically stable elements in limestones and marls indicates that the original clay mineral composition was rather homogeneous, which suggests no major difference in clay mineral input during “limestone times” and “marl times”. Even when plotting the data from the Frognerkilen Formation (where minor differences are observed in the palynomorph assemblages), we observe an excellent correlation between the lithologies, which is consistent with our interpretation that the cyclic signal in the abundances of a single species, *A. bornholmensis*, probably is not significant. The outlier XRF results we removed, from TC 12-203 (limestone) and TC 12-226 (marl) in Nakkholmen and WSA-12-090 (limestone) and WSA -12-074A (marl) in Bygdøy, represent a mix of both lithologies, so this does not influence the results.

In summary, in the dataset we collected, there is no evidence suggesting a primary rhythm. Nevertheless, as indicated in the introduction, this does not prove that these rhythmites are entirely of diagenetic origin. Differences may have existed that have been destroyed during diagenesis, or the proxies we measured may have been insensitive to the environmental cycles at work. For instance, chitinozoans do not respond directly to water depth or sea level changes (much less so than, e.g., acritarchs^39^) but the distribution of their assemblages is driven by SST^11^. So in order to observe a systematic difference in the palynomorph assemblages between limestone and marl beds, in a scenario where these reflect a glacial/interglacial climate signal, our sampling site, the Oslo-Asker District, would have needed to be positioned at a certain latitude, prone to be crossed by the shifting boundary between the latitudinal chitinozoan biotopes in every climate change cycle. If our zone of investigation stays within the same latitudinal biotope, even while it is contracting and expanding, those faunal variations will not be recorded in the cyclic sediment, and the environmental signal will not be clearly expressed. Nevertheless, during the time slabs investigated, the palaeolatitudinal position of the Oslo-Asker District shifted from approximately 50–45°S during the Early Ordovician to 30°S in the Late Ordovician^40^,^41^. The study site thus occupied the mid-latitudes during much of the Ordovician, which should constitute an appropriate position to detect polar front migrations during periods of extreme climate change, as we know from studies on the Pleistocene^42^ as well as on the Ordovician^11^,^33^.

A last aspect where chitinozoans may be useful to elucidate the nature of the rhythmites is as a high-resolution biostratigraphic marker. If the interpreted glacio-eustatic lows can be correlated to similar events in other epicratonic basins, this considerably strengthens the case for the rhythmites to be records of glaciations. Unfortunately, due to the poor preservation of the specimens recovered, we did not obtain sufficiently high-resolution data to make valid comments in this context for most of the rhythmites.

## Conclusion

In this study, we used different diagenetically inert parameters as a proxy to determine the origin of limestone-marl alternations, previously interpreted as the result of palaeoclimatic variations. Although the cyclicity is obvious in the outcrop, our data does not allow us to prove a primary origin of the signal, but rather suggests a diagenetic scenario: The micropaleontological analyses reveal no significant differences in palynomorph assemblages between the two lithologies, and the geochemical analyses show trend lines (linear fit) with similar slopes for limestones and marls, with a high correlation of diagenetically stable elements normalized to Al_2_O_3_. In the Frognerkilen Formation, one species seems to have different concentrations in each of the lithologies. However this result is considered insignificant in comparison to the overlarge majority of the palynological data, and it is not supported by the geochemical study. It is important to re-state, however, that lack of evidence for an original cyclic signal does not necessarily imply an entirely diagenetic origin of the rhythmites.

## Methods

### Stratigraphy and Sample location

**Figure 1:**
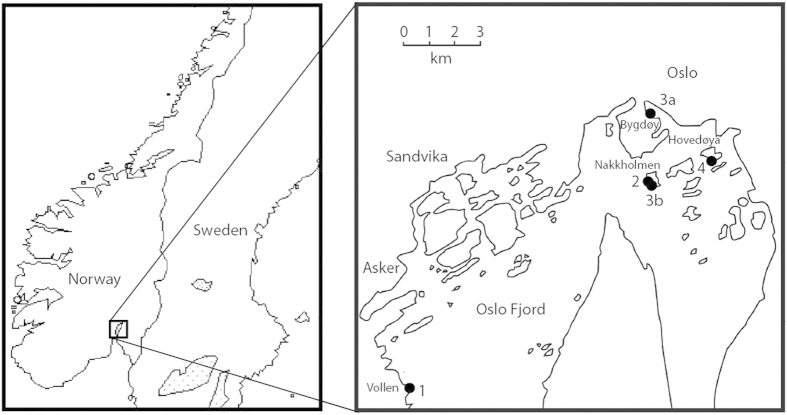
Map of the Oslo-Asker District showing the sampled locations. 1: Huk Formation in Vollen, 2: Arnestad and Frognerkilen formations on Nakkholmen Island, 3a Solvang Formation on the Bygdøy Peninsula, 3b: Solvang Formation on Nakkholmen Island, 4: Skogerholmen Formation on Hovedøya Island. Modified after Grahn *et al.*^49^.

**Figure 2:**
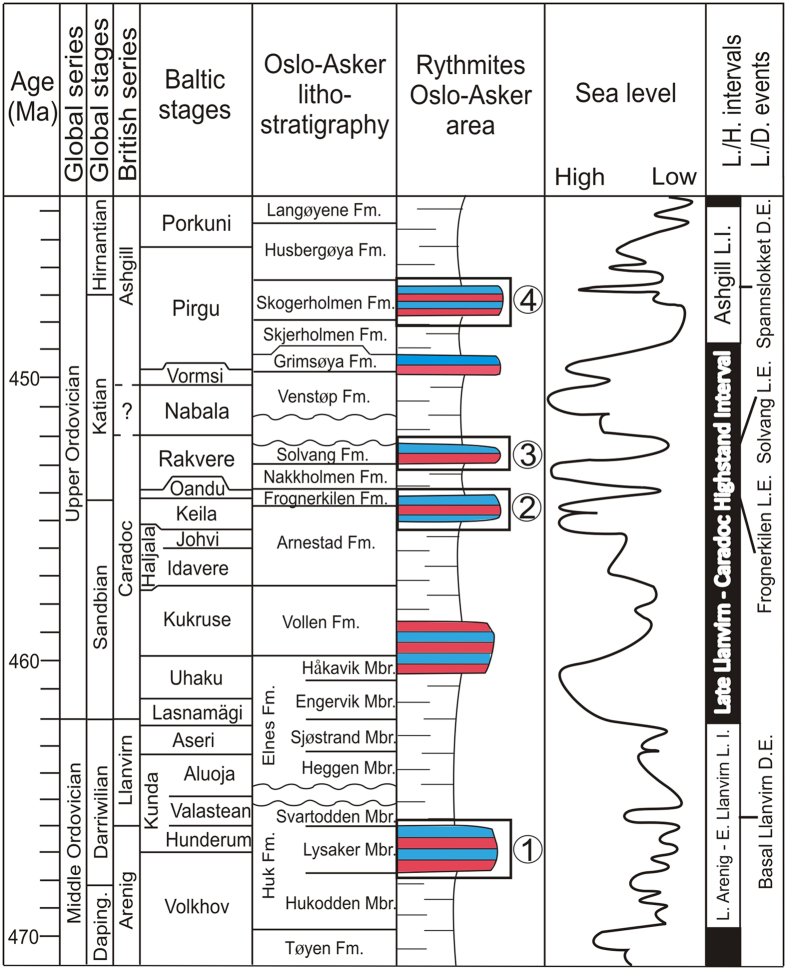
Lithostratigraphical chart of the Oslo-Asker District with the regional Baltic stages, the sea level curve and lowstand/highstand intervals (L.I/H.I) and lowstand/drowning events (L.E./D.E.) modified from Nielsen^27^. The black and white bars represent 2^nd^ order oscillations. The numbers refer to the four studied intervals corresponding to an interpreted lowstand event or interval. The red/blue alternations schematically represent the limestone/marl alternations.

**Figure 3:**
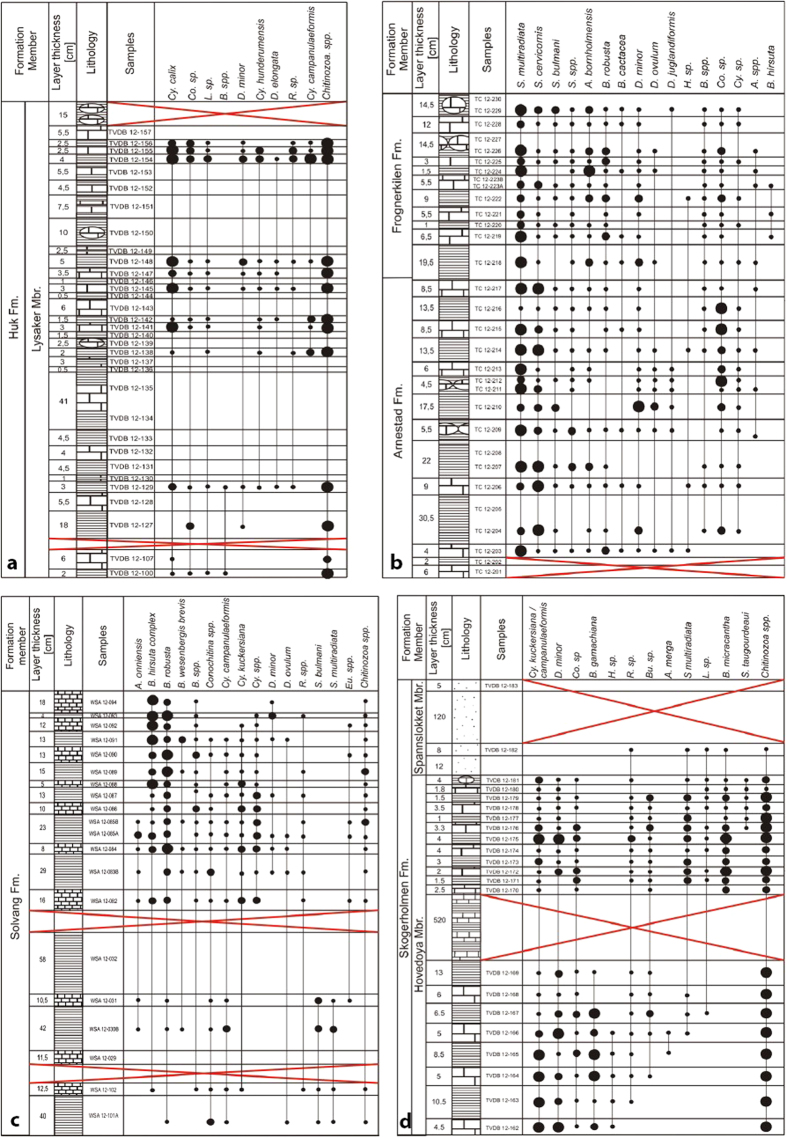
Stratigraphic log and palynomorph assemblages of the investigated sections. (**a**) Huk Section, (**b**) Nakkholmen Section, (**c**) Bygdøy Section, (**d**) Hovedøya Section. Small dots: <10 specimen per sample, medium dots: 10–40 specimen, big dots: >40 specimen.

**Figure 4:**
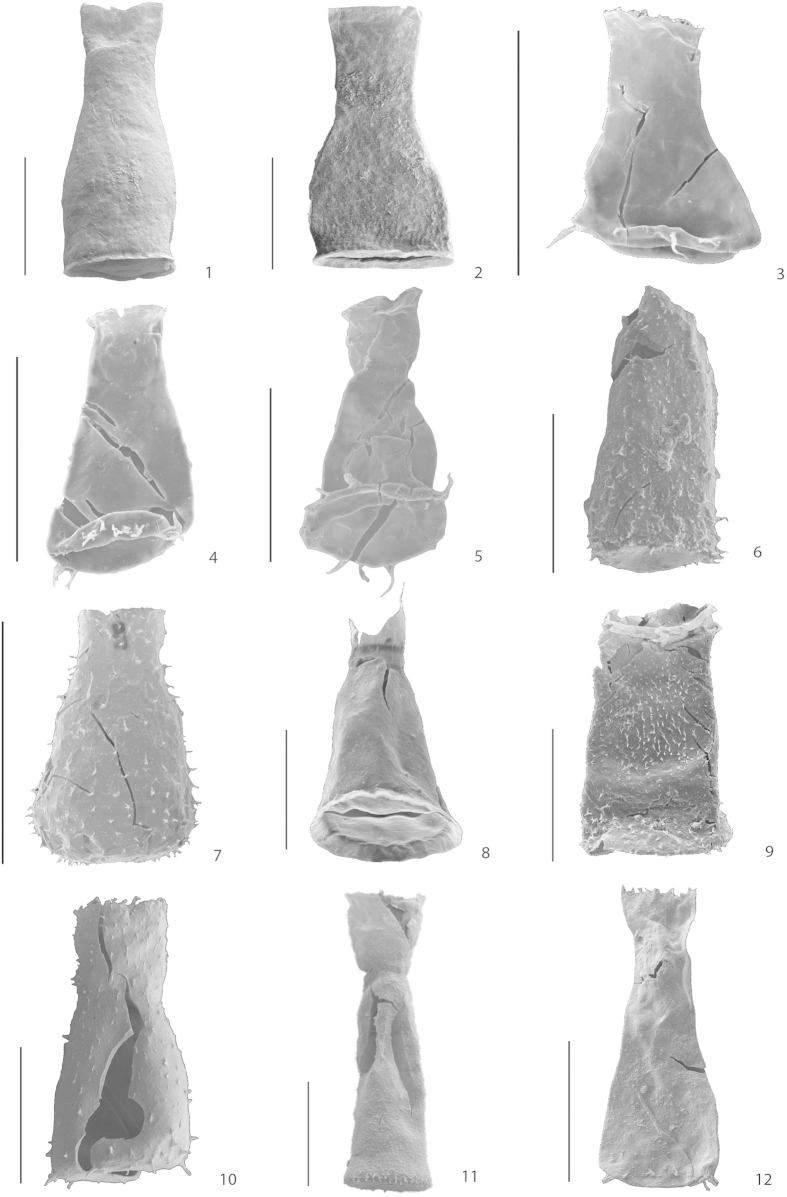
SEM images of selected chitinozoans. 1–2: Huk Formation. 1. *Cyathochitina calix*. 2. *Cyathochitina campanulaeformis*. 3–6: Arnestad/Frognerkilen formations. 3. *Ancyrochitina bornholmensis.* 4. *Spinachitina cervicornis*. 5. *Spinachitina multiradiata*. 6. *Belonechitina robusta*. 7–9: Solvang Formation: 7. *Belonechitina hirsuta complex*. 8. *Cyathochitina campanulaeformis*. 9. *Belonechitina robusta*. 10–12: Skogerholmen Formation: 10. *Belonechitina gamachiana*. 11. *Belonechitina micracantha*. 12. *Spinachitina* cf. *taugourdeaui*. Scale bar: 100 mμ.

**Figure 5:**
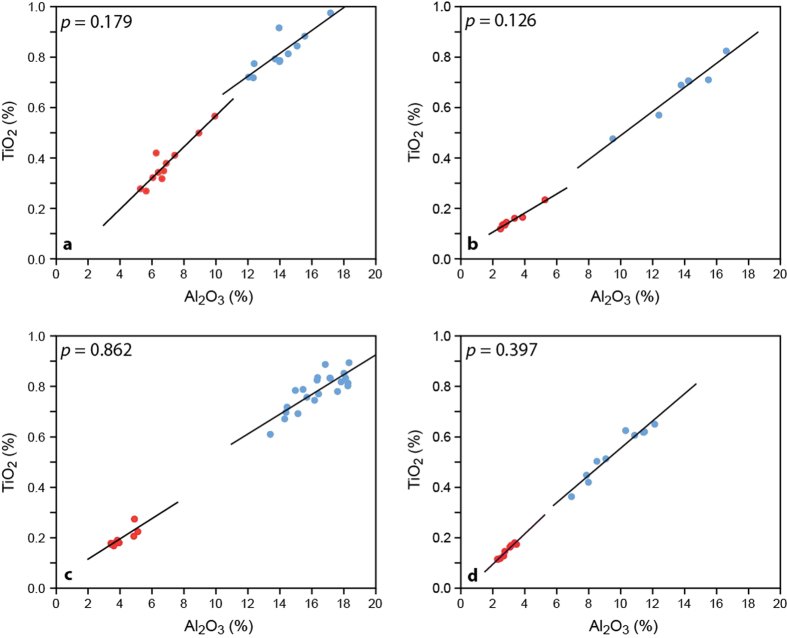
TiO_2_/Al_2_O_3_ data for: (a) Huk Formation, (b) Arnestad/Frognerkilen formations, (c) Solvang Formation, (d) Skogerholmen Formation. The data in each sections plot on regression lines with a high correlation between the two lithologies. Limestones are in red and marls in blue. *P* values <0.05 would indicate a significant difference between the slopes.

**Figure 6:**
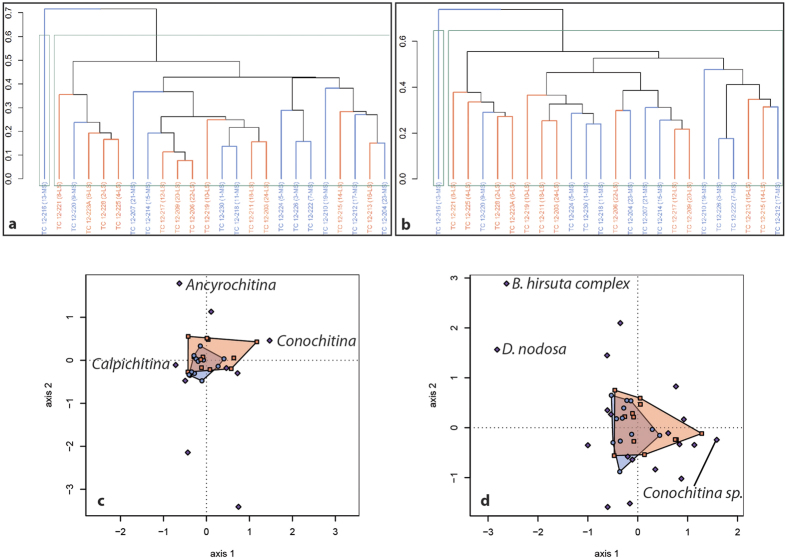
Statistical analyses for the chitinozoan assemblages of the Arnestad/Frognerkilen formations. (**a**) Hierarchical Cluster Analysis (Bray-Curtis index and UPGMA linkage) at the genus level shows no grouping. (**b**) Hierarchical Cluster Analysis at species level, showing no grouping, same as for genus level. (**c**) Detrended Correspondence Analysis (Bray-Curtis index) at genus level shows overlapping of the assemblage from both lithologies indicating a globally similar composition. (**d**) Detrended Correspondence Analysis at species level indicating a similar composition, same as for genus level. Limestones are in red and marls in blue.

During the Early Palaeozoic, the Oslo Oslo-Asker District^43^ (Fig. 1) was a cratonic basin on the Baltica palaeoplate^44^. A detailed review of its Ordovician stratigraphy by Owen *et al.*^14^ summarized the available data^45^,^46^,^47^. Nõlvak & Grahn^48^ and Grahn *et al.*^49^ gave a preliminary account of the chitinozoan biostratigraphy. The stratigraphy of the four studied intervals is summarized below:

1. The oldest interval comprises the Huk Formation and its three members^14^ (Fig. 2). The formation is about 6 m thick at the sampling location, and consists of massive limestones of the Hukodden Member and the Svartodden Member, bracketing the middle Lysaker Member, which is an alternation of nodular limestone and tectonised marl, and is the interval studied here. The formation ranges from the early Volkhov Baltic Stage to upper part of the Kunda Baltic Stage^50^. The Huk Formation was sampled and measured about one km south of Vollen (Fig. 1), at the junction of Elnesknausene road and Øvre Elnes Vei road (N59°47′49.2″ E10°29′17.9″).
2. The second interval comprises the Arnestad Formation^51^ – Frognerkilen Formation transition (Fig. 2). The former formation is 17.5 m thick and consists mainly of thick marl beds (30–40 cm) with only thin nodular or bedded limestone layers in between. It belongs to the Haljala and Keila Baltic stages^8^. The overlying Frognerkilen Formation^14^ comprises about 9.5 m of rubbly limestone changing upwards into nodular limestone and marls, and belongs to the Keila and Oandu Baltic stages^8^. This interval was measured and sampled at the southwestern edge of Nakkholmen Island in the Oslo Fjord (N59°53′17.48″ E10°41′29.17″) (Fig. 1).
3. The Solvang Formation^47^ (Fig. 2) has a thickness of 12 m at “Rødlokken Shore” and of 14.5 m on Nakkholmen Island, and belongs to the Rakvere Baltic Stage^8^. The Solvang Formation is developed as irregularly bedded and nodular limestone up to 20 centimeter thick with intervening calcareous marl layers that can be up to 60 cm in thickness. This third interval was mainly measured and sampled on the Bygdøy Peninsula, in a locality known as ‘Rodeløkken Shore’ (N59°54′54.07″ E10°41′28.92″) (Fig. 1.3a). Because the base of the formation is not well exposed at this location, a second section of the Solvang Formation was measured and sampled on the south-western part of Nakkholmen Island (N59°53′19.03″ E10°41′31.52″) (Fig. 1.3b). Two samples from the base of the formation from Nakkholmen Island complete the dataset from the ‘Rodeløkken Shore’.
4. The last interval comprises the Skogerholmen Formation^14^ (Fig. 2) and its two members. The formation consists of alternating limestone, marl and siltstone and is about 35 m thick and it represents the Pirgu Baltic Stage^8^. It comprises two members, i.e., the upper Spannslokket Member and the lower Hovedøya Member, the latter studied here. The Skogerholmen Formation was sampled and measured at the south-west corner of Hovedøya Island (N59°53′32.0″ E10°43′38.2″) (Fig. 1).

### Palynological analyses

The samples (50 g to 500 g) were collected bed-by-bed. The palynological analyses were carried out at the University of Lille (France) and Ghent University (Belgium). The protocol involves crushing the rock samples for a first acid treatment of 38% HCl during 24 hours. We used about 40 g for the marls (20 g in UGent) and 100 g for the limestones (30 g in UGent). The residue was then washed, prior to a second acid treatment with c. 200 ml 40–45% HF which was agitated (Lille) or heated to 80 °C (UGent) for 12 to 24 hours. Finally, the residues were washed with warm 38% HCl to remove any newly formed F-compounds before neutralization and filtering at 51 μm. The residues on the sieves were handpicked using a binocular microscope at 25–50 times magnification, and then identified with an SEM: a FEI Quanta 200 (Lille) and a JEOL JSM6400 (UGent).

The chitinozoan taxonomic composition was investigated using multivariate classification and ordination methods, as well as statistical tests. The dataset consists of abundance data (counts of chitinozoan specimens) in each sampled bed. First, a hierarchical cluster analysis (HCA) based on the Bray-Curtis similarity matrix and the UPGMA (Unweighted Pair Group Method with Arithmetic mean) linkage algorithm was performed to initially evaluate if the two lithologies cluster in two different groups or are mixed together. In the former case, this would indicate that the two lithologies have distinct taxonomic compositions. Alongside the HCA, a detrended correspondence analysis (DCA) has been applied. The purpose of this ordination method is to evaluate if some taxa are restricted to a specific lithology and consequently ordered along a gradient. The computed DCA was also based on the Bray-Curtis similarity matrix. Next, in order to have a statistical evaluation of the (dis)similarity of the chitinozoan taxonomic composition between the two lithologies, two multivariate tests have been performed: a one-way analysis of similarity (ANOSIM), and a one-way permutational multivariate analysis of variance (PERMANOVA). These tests were performed based on the Bray-Curtis similarity matrix, with 1000 permutations, and by setting the significance level at *p* = 0.05. Finally, an analysis of variance (ANOVA) has been performed to test whether the difference in mean abundance is statistically different between the two lithologies for each taxon separately. All these methods (HCA, DCA, ANOVA, ANOSIM, and PERMANOVA) are established methods for multivariate data analysis^52^,^53^,^54^,^55^,^56^. The descriptive, exploratory and statistical analyses of chitinozoan data have been performed using the environment R^57^ version 3.1.2 with the packages “vegan”^58^ and “epaleo” (C. Monnet, 2015, unpublished).The data analyses have been performed at two different taxonomic levels (genus and species), across the region as well as for each section separately. Due to significantly different sample sizes across the studied strata within and amongst the sections (chi square tests result into *p* < 0.001 for all sections and taxonomic levels), abundances of taxa within sampled beds were converted into relative proportions prior to the analyses.

### XRF analyses

In parallel, the samples were processed at the University of Erlangen for geochemistry. The samples were first crushed in a hydraulic press and finely ground and homogenized in a vibratory disc mill (Retsch RS 1, 50 ml agate grinding set). After drying in an oven at 105 °C for about 12 hours, 1.0006 g ± 0.6 mg of the sample was weighted in a porcelain crucible and stored in a muffle furnace at 1030 °C for 12 hours, pre-glowing the sample material, in order to measure the loss of ignition. The pre-glowed material was melted step-by-step with 4.830 g of lithium tetraborate (Li2B4O7) as a flux, and about 230 mg of di-iodine pentoxide (soldering flux), and the liquid was cast into a coquille. For the melting process, the manually homogenized compound was filled into platinum crucibles (98% Pt and 2% Au). The Oxiflux fusion system comprises three oxidation level/burners (450 °C, 550 °C, 650 °C) and two major burners (950 °C and 1050 °C). The quantitative major and trace elements analyses were carried out using a Spectro Xepos He energy-dispersive XRF spectrometer. The KH standard for limestone (Zentrales Geologisches Institut Berlin) and the JLs-1 standard for marls (Geological Survey of Japan) were used for XRF spectrometry. The XRF analyses allow us to test for diagenetic redistribution of major and/or trace elements. The elements that are part of the siliciclastic portion of the sediment, such as elements bound to clay minerals or heavy minerals like rutile, are less mobile than the calcareous components. When the calcite dissolution takes place, it results in a passive enrichment of insolubles like Ti or Al in the marl beds and a passive dilution of insolubles in the limestone beds. The concentrations of the major element (Si, Ti, Al, Fe, Mn, Mg, Ca, Na, K, P) and 12 trace elements (Ba, Cr, Ga, Nb, Ni, Pb, Rb, Sr, Th, V, Y, Zn, Zr) have been measured, specified as oxides (wt. %) for the major elements and single elements (ppm) for the trace elements. The elements are normalized relative to Al, because aluminum oxides do not dissolve at the usual pH and redox conditions during diagenetic alteration of deposits. The major and trace elements can be divided into three groups: the first group includes elements that are preferentially bound to clay minerals, such as Ti, Al, K, Rb and Si. Another group of elements (Mg, Fe, and Zn) can be identified by their preferential inclusion in the crystal lattice of calcite and clay minerals. A last group of elements are preferentially incorporated in the carbonates (Sr, Mn). The ratio between TiO_2_/Al_2_O_3_ is particularly appropriate to detect primary differences in the sediment because TiO_2_ shows a wide range for different clay minerals^26^. If the rhythmites record an environmental signal, we should observe trend lines with different slopes for the limestones and marls, whereas a similar slope suggests they can potentially emerge, diagenetically, from a single progenitor sediment^26^. The trend lines between the various elements and oxides normalized with Al_2_O_3_ have been computed by a linear fit with the major axis approach for limestones and marls separately. The difference in the slopes of these trend lines have been assessed statistically using the maximum likelihood estimate for the common slope and the Bartlett-corrected likelihood ratio test^59^, as implemented in the software PAST^60^ version 3.09.

## Additional Information

**How to cite this article**: Amberg, C. E. A. *et al.* The nature of Ordovician Limestone-marl alternations in the Oslo-Asker District (Norway): witnesses of primary glacio-eustasy or diagenetic rhythms? *Sci. Rep.* **6**, 18787; doi: 10.1038/srep18787 (2016).

## Supplementary Material

Supplementary Information
