# Supplementary material for: The nature of Ordovician limestone-marl alternations in the Oslo-Asker District (Norway): witnesses of primary glacio-eustasy or diagenetic rhythms?
Source: Sci Rep. 2016 Jan 7;6:18787. doi: 10.1038/srep18787 (PMC4704048; doi:10.1038/srep18787)
Supplement: Supplementary Information [file srep18787-s1.doc]

**Supplementary information for:**

**The nature of Ordovician Limestone-mudstone alternations in the Oslo-Asker area (Norway): witnesses of primary glacio-eustasy or diagenetic rhythms?**

Chloé E. A. Amberg, Tim Collart, Wout Salenbien, Lisa M. Egger, Axel Munnecke, Arne T. Nielsen, Claude Monnet, Øyvind Hammer & Thijs R. A. Vandenbroucke.


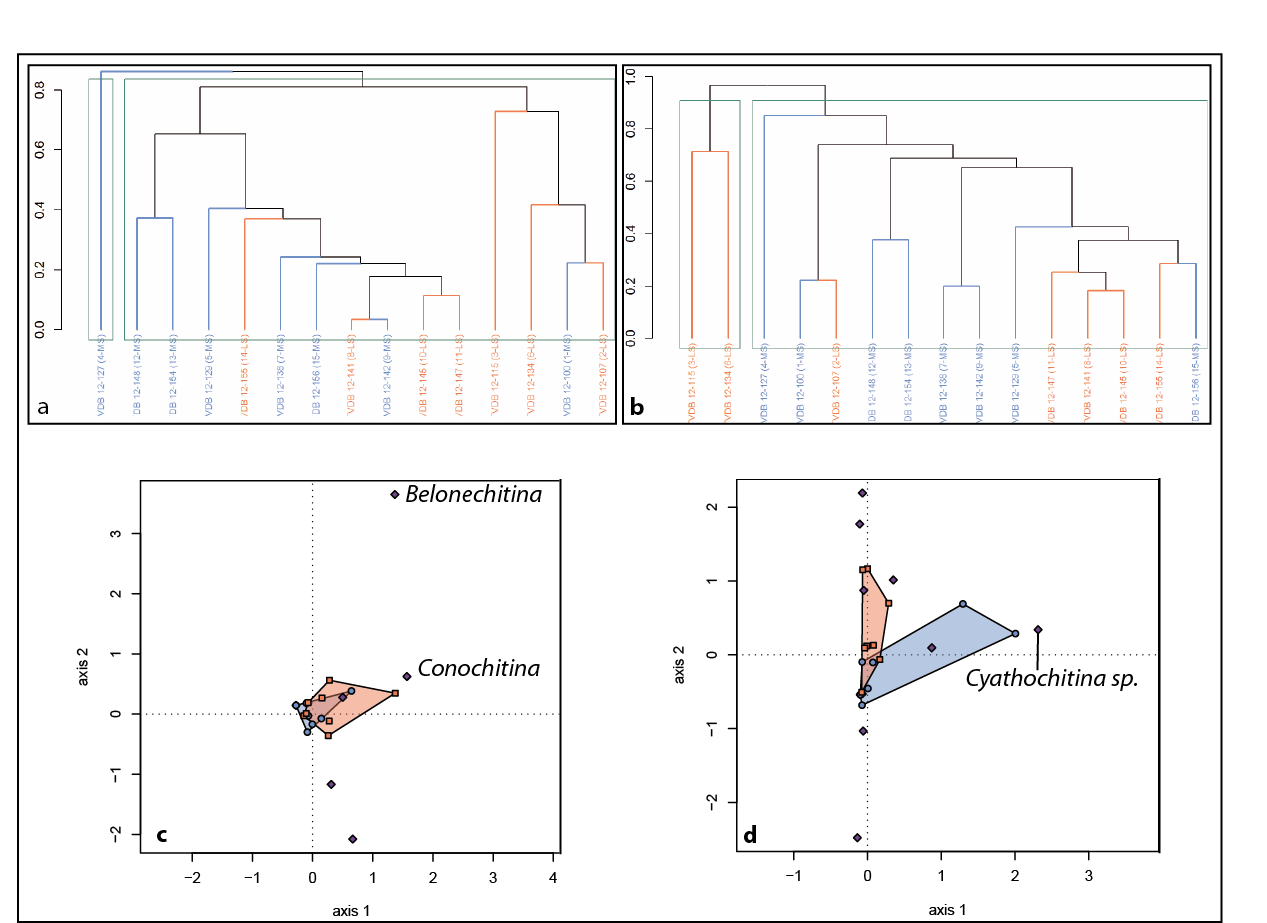


**Supplementary Figure S1.** Statistical analysis for the chitinozoan assemblages of the Lysaker Member of the Huk Formation. (a) Hierarchical Cluster Analysis (Bray-Curtis index and UPGMA linkage) at genus level. (b) Hierarchical Cluster Analysis at species level. (c) Detrended Correspondence Analysis (Bray-Curtis index) at genus level. (d) Detrended Correspondence Analysis at species level. The analyses indicate a similar composition of assemblages. Limestones are in red, mudstones in blue.


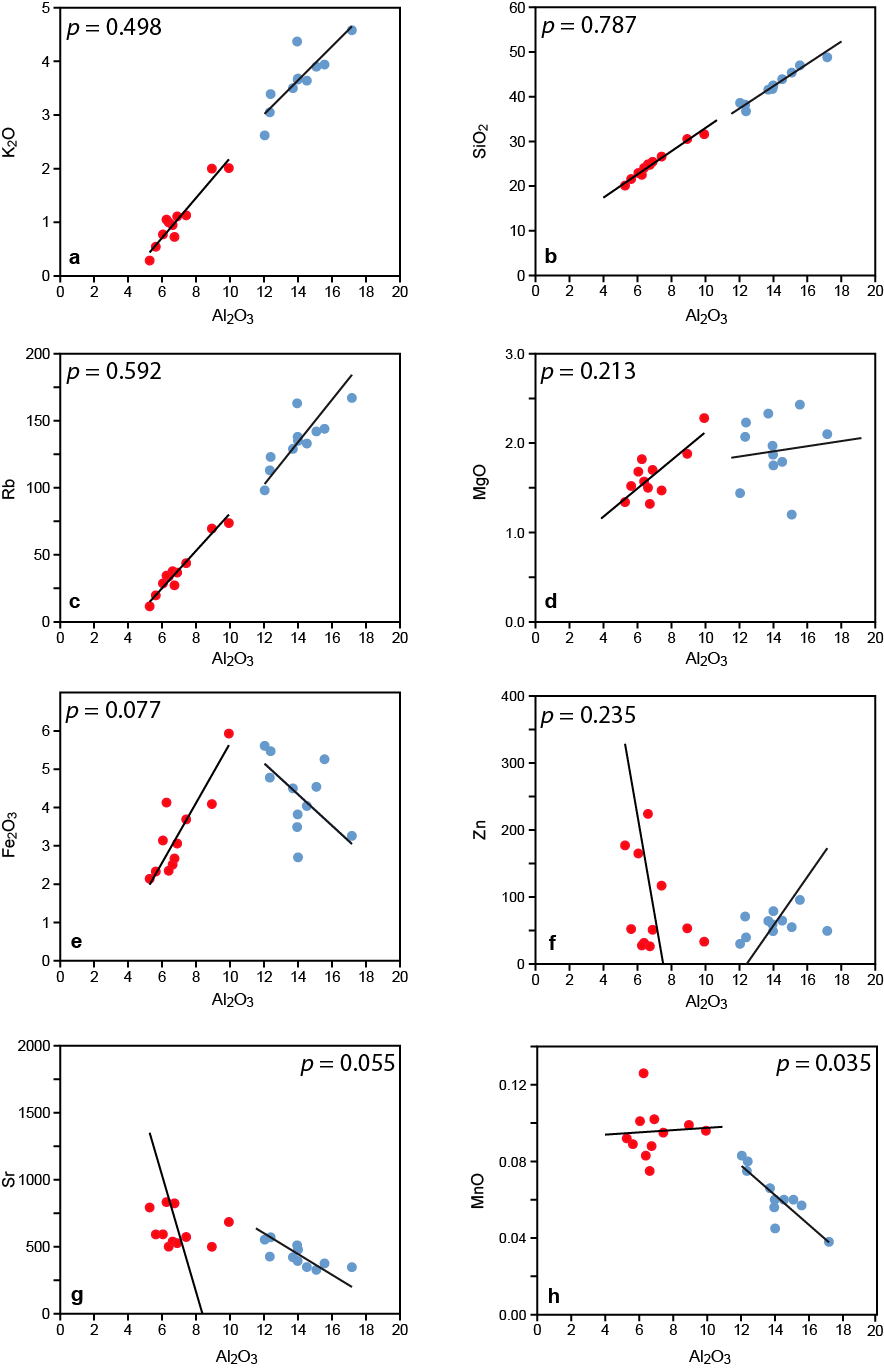


**Supplementary Figure S2.** XRF element ratios for the Lysaker Member of The Huk Formation. a-c: Elements preferentially bound to clay minerals. d-f: Elements preferentially bound to clay and calcite minerals. g-h: Elements bound to calcite minerals. Limestones are in red and mudstones in blue. *P* values < 0.05 indicate significant difference between the slopes.

| **Taxa** | **F** | ***P*** |
| --- | --- | --- |
| *Ancyrochitina* | 6.21 | 0.015 |
| *Ancyrochitina bornholmensis* | 4.19 | 0.044 |
| *Conochitina* | 3.25 | 0.075 |
| *Belonechitina hirsuta* | 2.95 | 0.090 |
| *Conochitina sp* | 2.80 | 0.098 |
| *Rhabdochitina* | 2.59 | 0.111 |
| *Rhabdochitina sp* | 2.59 | 0.111 |
| *Ancyrochitina onniensis* | 2.19 | 0.143 |
| *Lagenochitina* | 2.17 | 0.145 |
| *Lagenochitina sp* | 2.17 | 0.145 |
| *Desmochitina ovulum* | 2.05 | 0.156 |
| *Belonechitina wesenbergis brevis* | 1.73 | 0.192 |
| *Desmochitina nodosa* | 1.61 | 0.208 |
| *Belonechitina cactacea* | 1.60 | 0.210 |
| *Euconochitina* | 1.48 | 0.228 |
| *Euconochitina sp* | 1.48 | 0.228 |
| *Bursachitina* | 1.28 | 0.261 |
| *Bursachitina sp* | 1.28 | 0.261 |
| *Calpichitina* | 1.27 | 0.263 |
| *Calpichitina sp* | 1.27 | 0.263 |
| *Ancyrochitina sp* | 1.00 | 0.320 |
| *Belonechitina hirsuta complex* | 0.87 | 0.353 |
| *Cyathochitina* | 0.86 | 0.357 |
| *Cyathochitina calix* | 0.82 | 0.367 |
| *Spinachitina bulmani* | 0.74 | 0.392 |
| *Desmochitina* | 0.42 | 0.519 |
| *Belonechitina* | 0.38 | 0.538 |
| *Belonechitina robusta* | 0.31 | 0.580 |
| *Spinachitina sp* | 0.23 | 0.634 |
| *Desmochitina minor* | 0.21 | 0.645 |
| *Cyathochitina huderumensis* | 0.18 | 0.674 |
| *Cyathochitina gr.campanulaeformis/kuckersiana* | 0.12 | 0.733 |
| *Desmochitina juglandiformis* | 0.10 | 0.745 |
| *Conochitina minnesotensis* | 0.10 | 0.755 |
| *Belonechitina gamachiana* | 0.06 | 0.811 |
| *Belonechitina micracantha* | 0.05 | 0.820 |
| *Spinachitina* | 0.04 | 0.840 |
| *Desmochitina elongata* | 0.03 | 0.849 |
| *Hercochitina* | 0.03 | 0.853 |
| *Hercochitina sp* | 0.03 | 0.853 |
| *Spinachitina multiradiata* | 0.03 | 0.869 |
| *Spinachitina cf. taugourdeaui* | 0.01 | 0.919 |
| *Belonechitina sp* | 0.01 | 0.921 |
| *Cyathochitina sp* | 0.00 | 0.950 |
| *Spinachitina cervicornis* | 0.00 | 0.958 |

**Supplementary Fig. S3**: One-way ANOVA of each taxon separately in order to test for their equality of mean relative abundance between the two lithologies (37 samples of limestones and 39 of mudstones).


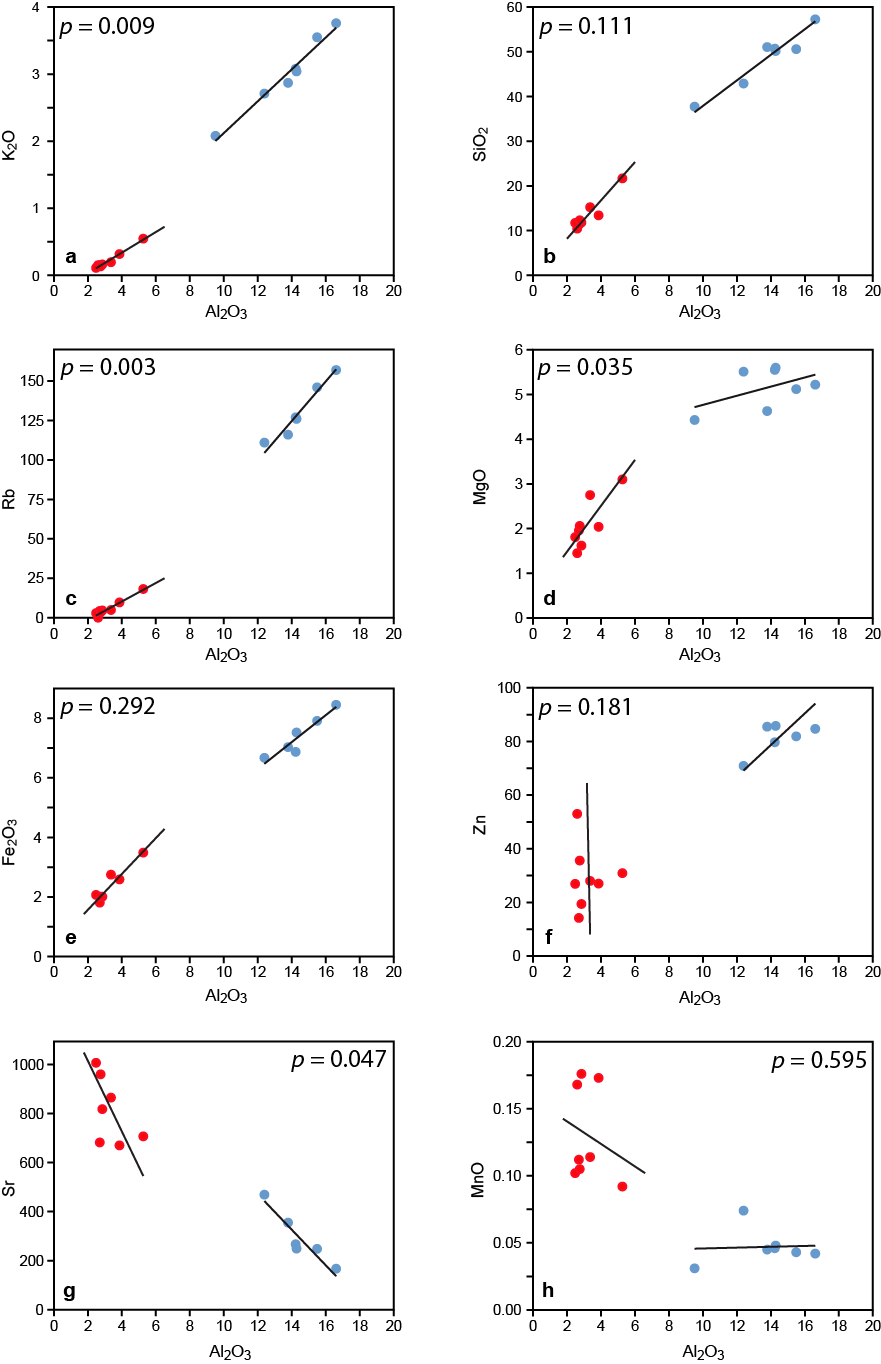


**Supplementary Figure S4.** XRF element ratios for Arnestad/Frognerkilen Formation. a-c: Elements preferentially bound to clay minerals. d-f: Elements preferentially bound to clay and calcite minerals. g-h: Elements bound to calcite minerals. Limestones are in red and mudstones in blue. *P* values < 0.05 indicate significant difference between the slopes.


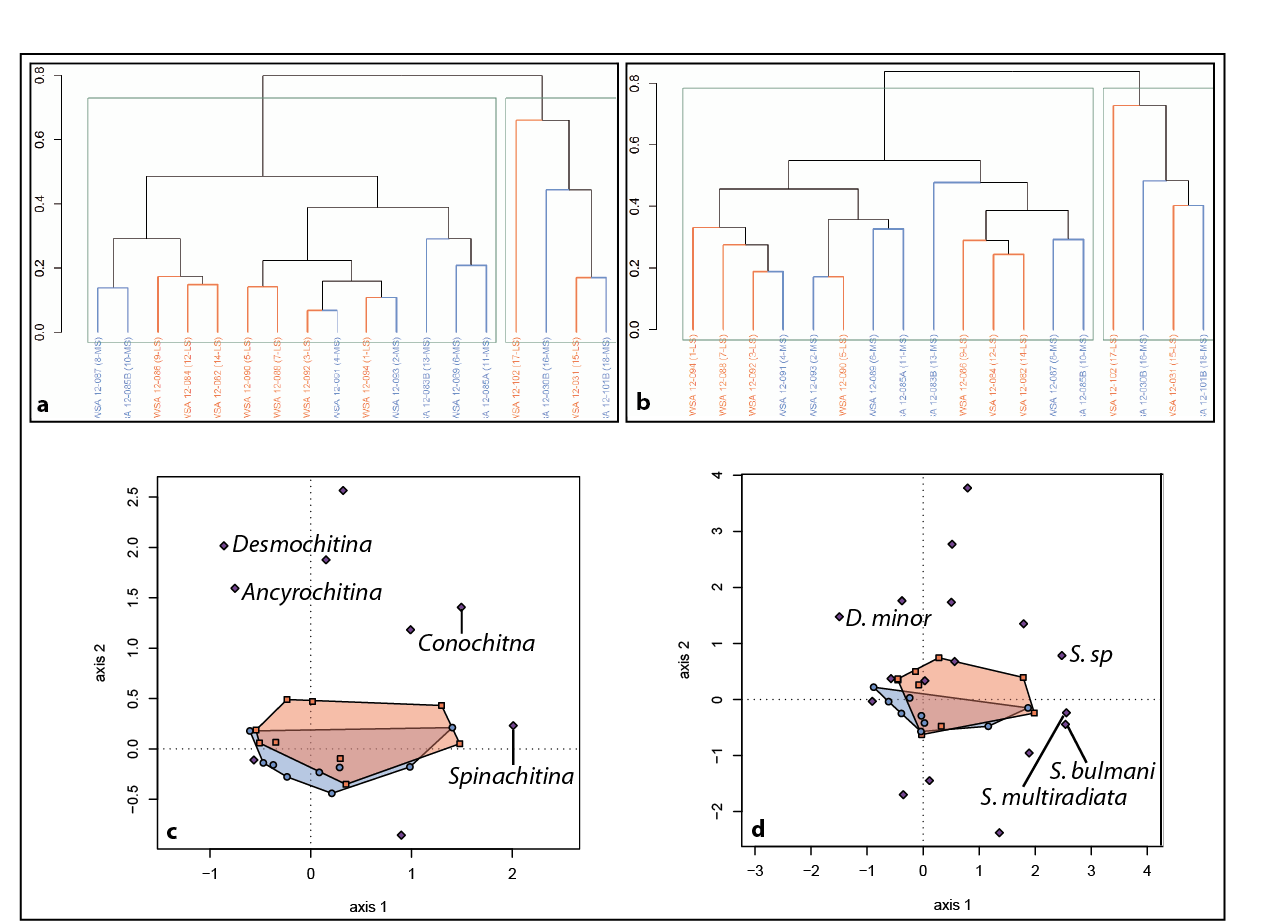


**Supplementary Figure S5.** Statistical analysis for the chitinozoan assemblage of the Solvang Formation. (a) Hierarchical Cluster Analysis (Bray-Curtis index and UPGMA linkage) at genus level. (b) Hierarchical Cluster Analysis (Bray-Curtis index and UPGMA linkage) at species level. (c) Detrended Correspondence Analysis (Bray-Curtis index) at genus level. (d) Detrended Correspondence Analysis (Bray-Curtis index) at species level. The statistical analyses indicate a similar composition of assemblages. Limestones are in red, mudstones in blue.


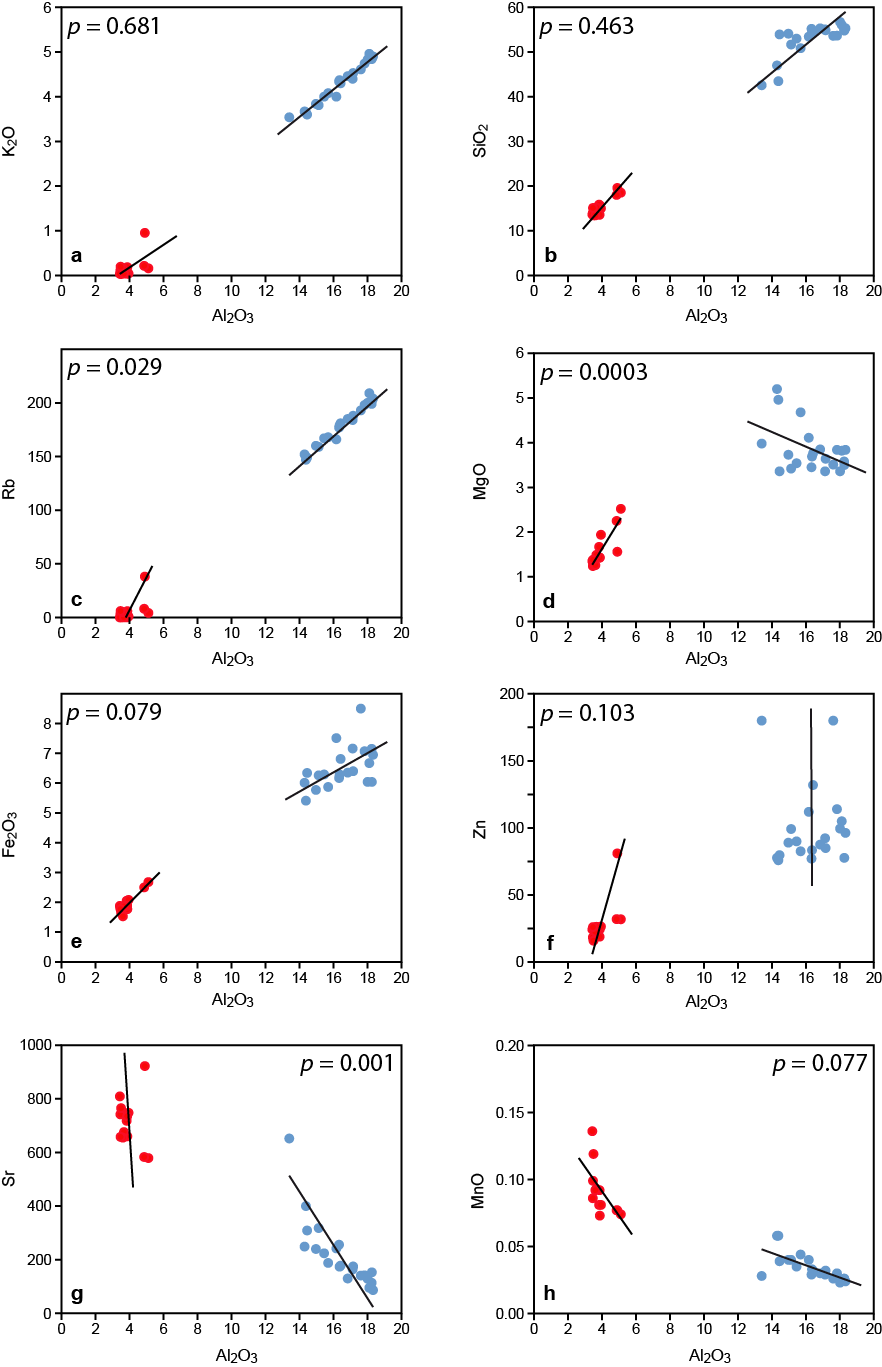


**Supplementary Figure S6.** XRF element ratios for the Solvang Formation. a-c: Elements preferentially bound to clay minerals. d-f: Elements preferentially bound to clay and calcite minerals. g-h: Elements bound to calcite minerals. Limestones are in red and mudstones in blue. *P* values < 0.05 indicate significant difference between the slopes.


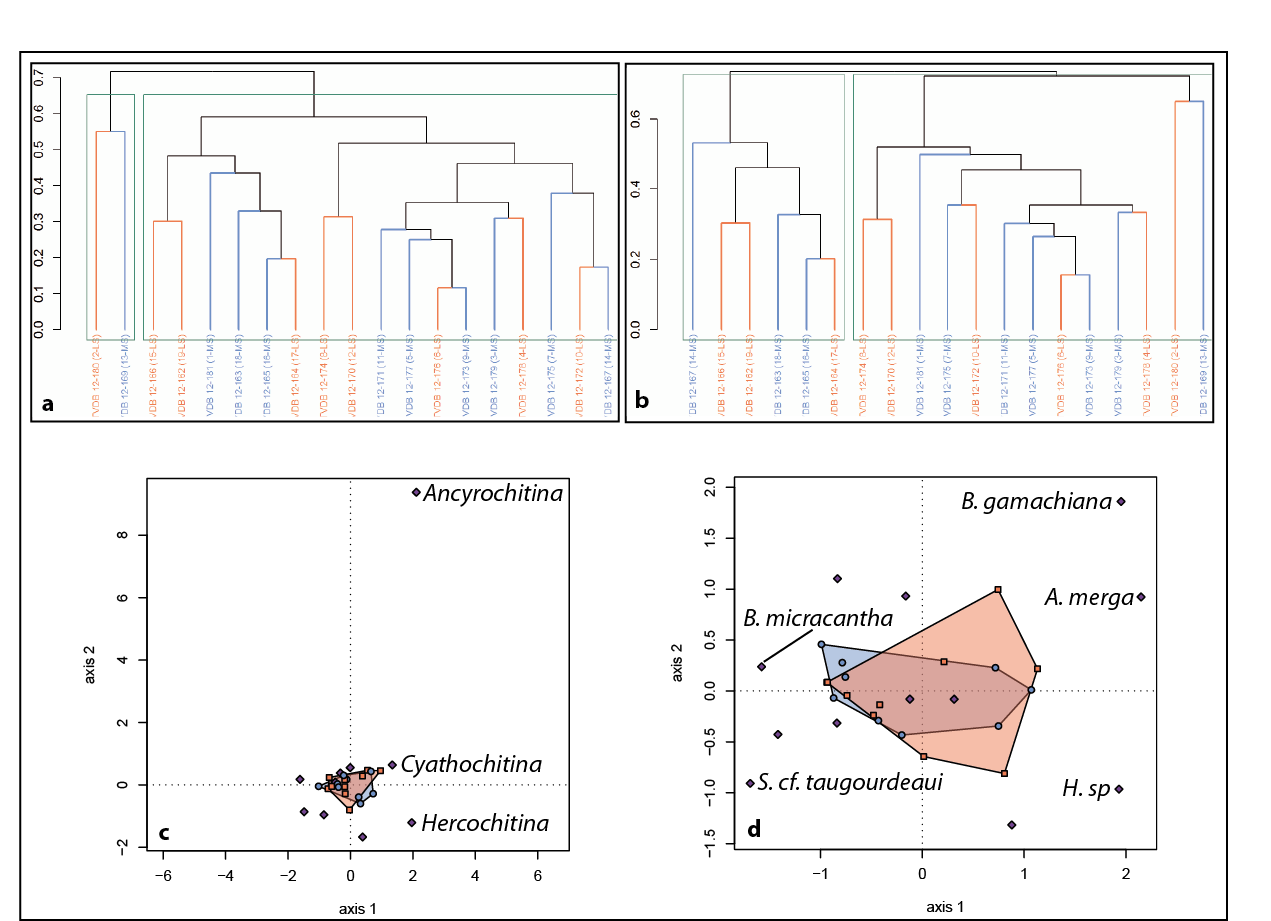


**Supplementary Figure S7.** Statistical analysis for the Hovedøya Member of the Skogerholmen Formation. (a) Hierarchical Cluster Analysis (Bray-Curtis index and UPGMA linkage) at genus level. (b) Hierarchical Cluster Analysis (Bray-Curtis index and UPGMA linkage) at species level (c) Detrended Correspondence Analysis (Bray-Curtis index) at genus level. (d) Detrended Correspondence Analysis (Bray-Curtis index) at species level. The statistical analyses indicate a similar composition of assemblages. Limestones are in red, mudstones in blue.


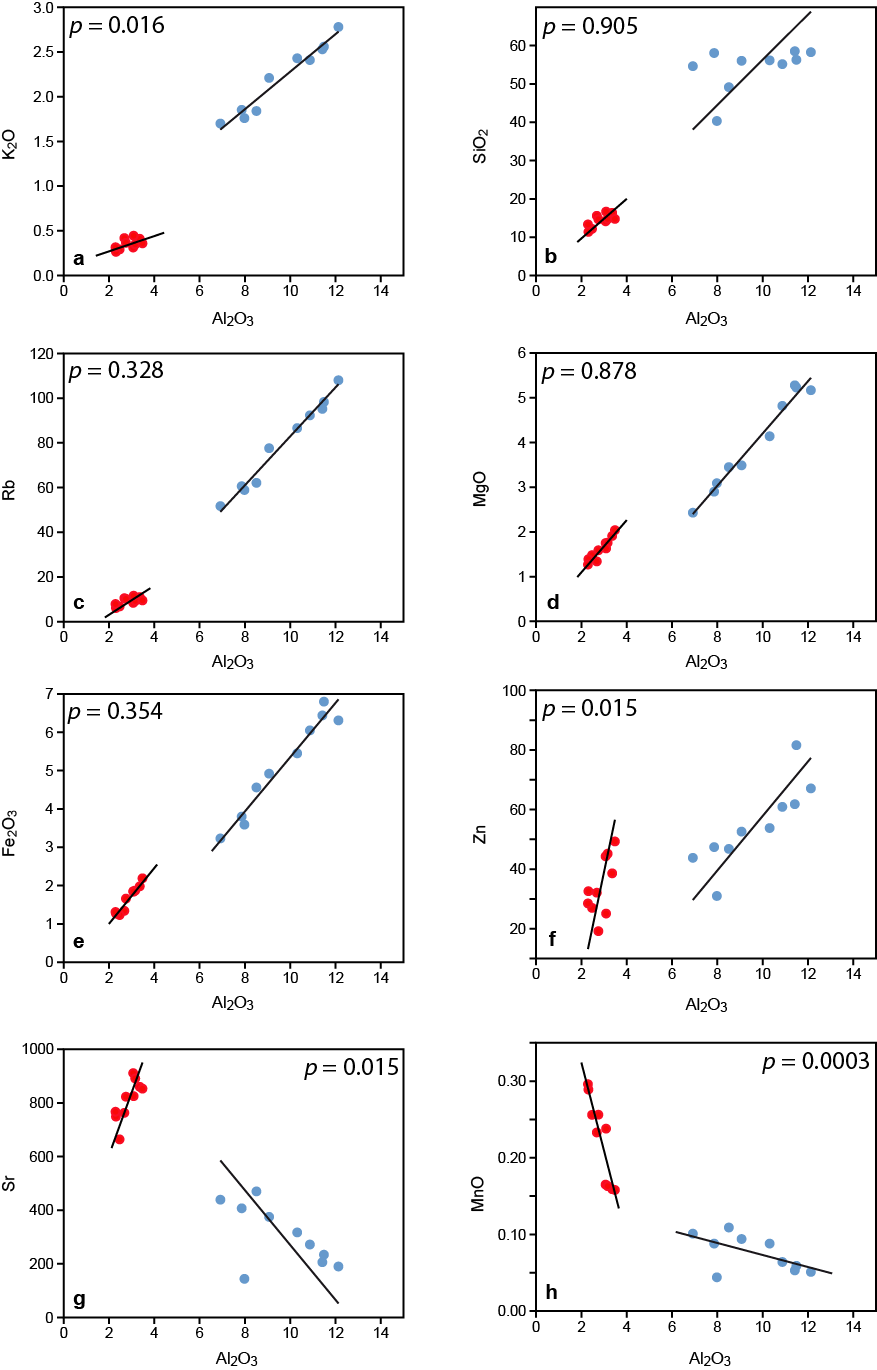


**Supplementary Figure S8.** XRF element ratios for the Hovedøya Member of the Skogerholmen Formation. a-c: Elements preferentially bound to clay minerals. d-f: Elements preferentially bound to clay and calcite minerals. g-h: Elements bound to calcite minerals. Limestones are in red and mudstones in blue. *P* values < 0.05 indicate significant difference between the slopes.
